# FAST INdiCATE Trial protocol. Clinical efficacy of functional strength training for upper limb motor recovery early after stroke: Neural correlates and prognostic indicators

**DOI:** 10.1111/ijs.12179

**Published:** 2013-09-12

**Authors:** Valerie M Pomeroy, Nick S Ward, Heidi Johansen-Berg, Paulette van Vliet, Jane Burridge, Susan M Hunter, Roger N Lemon, John Rothwell, Christopher J Weir, Alan Wing, Andrew A Walker, Niamh Kennedy, Garry Barton, Richard J Greenwood, Alex McConnachie

**Affiliations:** 1 Faculty of Medicine and Health Sciences, University of East AngliaNorwich, UK; 2 Institute of Neurology, University College LondonLondon, UK; 3 Nuffield Department of Clinical Neuroscience, University of Oxford, John Radcliffe HospitalOxford, UK; 4 School of Health Sciences, University of NewcastleNewcastle, NSW, Australia; 5 Faculty of Health Sciences, University of SouthamptonHighfield, UK; 6 Institute for Science and Technology in Medicine, Keele UniversityKeele, UK; 7 Medical Research Council Hub for Trials Methodology Research, Centre for Population Health Sciences, University of EdinburghEdinburgh, UK; 8 School of Psychology, University of BirminghamBirmingham, UK; 9 School of Medicine, University of East AngliaNorwich, UK; 10 Robertson Centre for Biostatistics, University of GlasgowGlasgow, UK

**Keywords:** functional strength training, movement performance therapy, neuroimaging, physical therapy, rehabilitation, stroke, upper limb

## Abstract

**Rationale:**

Functional strength training in addition to conventional physical therapy could enhance upper limb recovery early after stroke more than movement performance therapy plus conventional physical therapy.

**Aims:**

To determine (a) the relative clinical efficacy of conventional physical therapy combined with functional strength training and conventional physical therapy combined with movement performance therapy for upper limb recovery; (b) the neural correlates of response to conventional physical therapy combined with functional strength training and conventional physical therapy combined with movement performance therapy; (c) whether any one or combination of baseline measures predict motor improvement in response to conventional physical therapy combined with functional strength training or conventional physical therapy combined with movement performance therapy.

**Design:**

Randomized, controlled, observer-blind trial.

**Study:**

The sample will consist of 288 participants with upper limb paresis resulting from a stroke that occurred within the previous 60 days. All will be allocated to conventional physical therapy combined with functional strength training or conventional physical therapy combined with movement performance therapy. Functional strength training and movement performance therapy will be undertaken for up to 1·5 h/day, five-days/week for six-weeks.

**Outcomes and Analysis:**

Measurements will be undertaken before randomization, six-weeks thereafter, and six-months after stroke. Primary efficacy outcome will be the Action Research Arm Test. Explanatory measurements will include voxel-wise estimates of brain activity during hand movement, brain white matter integrity (fractional anisotropy), and brain–muscle connectivity (e.g. latency of motor evoked potentials). The primary clinical efficacy analysis will compare treatment groups using a multilevel normal linear model adjusting for stratification variables and for which therapist administered the treatment. Effect of conventional physical therapy combined with functional strength training versus conventional physical therapy combined with movement performance therapy will be summarized using the adjusted mean difference and 95% confidence interval. To identify the neural correlates of improvement in both groups, we will investigate associations between change from baseline in clinical outcomes and each explanatory measure. To identify baseline measurements that independently predict motor improvement, we will develop a multiple regression model.

## Introduction

This trial addresses an important focus for stroke rehabilitation: the ability of stroke survivors to recover the use of their arm and hand for everyday functional tasks such as picking up a cup, unscrewing a coffee jar lid, and doing up buttons and zippers. Limitations in performing such everyday tasks seriously affect capacity for independent living. At six-months after stroke, only 38% of people who receive rehabilitation have recovered some dexterity 1.

Systematic reviews [for example 2] indicate that repetitive functional task-specific activity can enhance motor recovery. Two training modalities that have attracted considerable attention are constraint-induced movement therapy (CIMT), which consists of repetitive practice of functional tasks combined with constraint of the ipsilesional upper limb, and robot-assisted therapy (RAT), which provides repetitive practice of movements that are required for upper limb functional activity. CIMT is effective between three- and nine-months after stroke 3 but less so when given early after stroke 4. Furthermore, CIMT is suitable only where at least 10 degrees of active movement are present in the paretic thumb and two or more paretic fingers 5. This requirement for a high level of function excludes many stroke survivors 6. RAT has been used in a wider group of stroke survivors but has also been found to be no more effective than an equal dose of conventional therapy 7. Thus, the question of whether a novel treatment aimed at enhancing upper limb functional task-specific activity might be better than routine conventional physical therapy (CPT) remains unanswered.

Functional strength training (FST) combines functional task-specific exercise and strength training, the latter of which is included because the largest impact on upper limb functional recovery after stroke may result from loss of muscle strength 8. Importantly, preliminary data indicate FST may be more effective than another therapy, movement performance therapy (MPT), at equal intensity 9. Both FST and MPT are used in routine CPT.

Evaluation of FST through properly designed clinical trials is crucial. We are aware, however, that a potentially serious barrier to the development of any novel treatment for poststroke motor impairment arises from poor understanding of its mechanisms of action and, in particular, whether it is likely to work in all stroke sub-groups 10. The CIMT and RAT trials recruited patients based on clinical phenotype, but in order to target therapy to those most likely to benefit, this may not be sufficient. It is increasingly recognized that large rehabilitation trials need to include more sophisticated measures of residual brain structure and function in order for researchers to understand the mechanisms of action of treatment and the characteristics of ‘responsive’ patients 10,11. For example, both the pretreatment level of brain activity in primary motor cortex during the performance of a motor task 12 and the degree of damage to descending motor white matter pathways 13 were associated with greater clinical improvement in 24 chronic stroke patients undergoing two-weeks of robot-based therapy. These studies are encouraging, but if we are to incorporate such data into models that can accurately predict therapeutic response, larger sample sizes are clearly required.

Specific objectives of this trial are the following:

1. to determine whether CPT + FST commenced early after stroke produces greater improvements in upper limb motor recovery than CPT + MPT;
2. to identify similarities and differences in the neural correlates of clinical improvement in response to (a) CPT + FST and (b) CPT + MPT;
3. to determine whether any pretreatment parameters or combinations are sufficiently predictive of improvement in upper limb motor function in response to intervention to enable physical therapy to be targeted at those most likely to respond;
4. to provide estimates of the preliminary cost-effectiveness of CPT + FST and inform the design of a subsequent pragmatic trial.

## Methods

### Design

**Figure 1:**
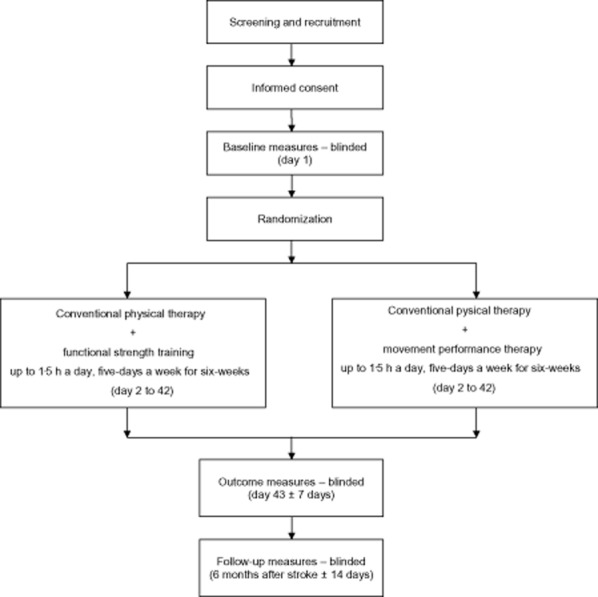
Flow diagram to illustrate trial design.

The trial will be a randomized, controlled, observer-blind trial (Fig. 1).

### Study population

Study criteria (combined inclusion and exclusion) are as follows:

- aged 18 + years;
- infarction in anterior cerebral circulation territory within the previous 60 days, confirmed by clinical neuroimaging;
- score at least 11/33 for Motricity Index pinch section 14 but unable to complete the nine-hole peg test (9HPT) 14 in 50 s or less;
- no obvious spatial neglect as defined by a score of 0 or 1 on Extinction and Inattention sub-scale of the NIH Stroke Scale;
- no obvious motor dyspraxia or communication deficits as assessed by ability to imitate action with the nonparetic upper limb, as in previous pilot work 15;
- able, prior to the index stroke, to use the paretic upper limb to lift a cup and drink from it.

### Randomization

The randomization sequence, generated in advance, will stratify participants by clinical center, time after stroke (up to 30 days and 31–60 days), and ability to use the paretic upper limb as assessed by the 9HPT 14 (substantial impairment = able to move one peg or less in 50 s; moderate impairment = able to move two or more pegs in 50 s). An independent telephone randomization service will maintain allocation concealment from the research team prior to randomization. An independent statistician at the Robertson Centre will generate the randomization sequence and will not be involved in developing the statistical analysis plan or programming the trial statistical analysis.

### Interventions

The intervention phase will last for six-weeks. All participants will receive routine CPT provided by the clinical physiotherapists, as in previous pilot work 9. Training will be provided for them to record content and amount of therapy provided daily for home participants, outpatients, and inpatients.

In each clinical center, the extra therapy will be provided by a research therapist responsible for either extra MPT (control) or extra FST (experimental). We will not tell clinical staff which research therapist is responsible for which treatment. This strategy is expected to minimize the potential for therapist bias, although allocation to different forms of exercise-based therapy is more difficult to conceal than, for example, an active or placebo drug, because of observable differences.

Participants in both groups will take part in extra therapy, as prescribed and overseen by a research therapist through direct and nondirect contact, for up to 1·5 h a day, five-days a week for six-weeks. The research therapists will be trained to direct either FST or MPT in accordance with a standardized manual before the trial begins. Fidelity to the manuals will be assessed at the beginning and at regular points throughout the trial with little prior warning to therapists.

### Control intervention (CPT + MPT)

MPT is the component of CPT which emphasizes movement quality rather than quantity. It is based on neurophysiological approaches for which there is evidence that they produce no effect compared with no treatment or a placebo 16. MPT involves joint and soft tissue mobilization, sensory stimulation, facilitation of muscle activity/movement, positioning, retraining normal patterns of movement, and education for the patient/carer. Emphasis is given to hands-on interventions provided by a therapist facilitating and guiding movement (therapist-dependent) to provide sensory input and to optimize postural control and joint alignment in preparation for voluntary movement. Some repetitive practice of functional tasks is included, but without systematic progression in resistance to movement.

Feedback and instructions during MPT encourage an internal focus of attention on the movement performance, for example, amount and direction of elbow movement when lifting a teapot.

### Experimental intervention (CPT + FST)

FST involves repetitive progressive resistive exercise during goal-directed functional activity, with the therapist providing verbal prompting and feedback (therapist-independent). FST is based on the key elements of normal upper limb function (i.e., positioning the hand and then using it to manipulate objects) and is therapist-independent while maintaining participant safety. The focus is on improving the power of shoulder/elbow muscles to enable appropriate placing of the hand, improving production of appropriate force in arm and hand muscles to achieve a specific grasp, and specific resistive functional practice for wrist and finger muscles to maximize ability to manipulate objects. The initial level of load/resistance is the maximum load that still permits five repetitions of action through the available range of muscle length. Treatment is progressed systematically using repetition and increased resistance to movement by changing the limb’s relationship to gravity, changing the amount of friction to overcome, and increasing the size and weight of items (e.g., following an empty cup with a cup containing an increased volume of water). FST involves specific movements for muscle groups, upper limb gross movement patterns underlying functional activity, hand reaching/retrieval activities, hand grip activities, hand manipulation involving entire everyday movements, and using objects such as screw-top canisters, pegs, mugs, and pens. These movements are extended into more complex everyday activities such as placing different food items into a shopping bag, then lifting the bag onto a shelf; tightening/loosening nuts/bolts; opening a bottle and drinking from it; and pouring tea from a pot.

Feedback and instructions encourage an external focus of attention on the effects of movements, for example, whether the teapot has been lifted clearly off the table.

### Outcomes

In accordance with the intention-to-treat principle, every effort will be made to include all randomized participants at outcome and follow-up whether or not they discontinue randomized treatment before the end of the intervention phase. Measurements will be made and processed by assessors blinded to treatment allocation. To assess whether blinding was achieved, we will ask assessors, at outcome and follow-up points, to guess participants’ group allocation. Agreement with actual allocation will be assessed with Cohen’s kappa.

**Clinical efficacy measures** will be made pre-randomization (baseline), the day (± 7) after the six-week intervention phase ends (outcome), and six calendar months (± 14 days) after the index stroke (follow-up).

The primary outcome measure will be the score on the Action Research Arm Test (ARAT) 17, a measure of the primary focus of both interventions – improved upper limb function.

Secondary outcome measures will be (a) Wolf Motor Function Test (WMFT) score 18, (b) hand grip force, and (c) pinch grip force. The upper limb positions for both pinch grip force and hand grip force will be standardized and the myometer set to zero after the participant’s hand/digits are positioned around the bars, ‘at rest’. The instruction given will be ‘squeeze as hard as you can’. Force values will be obtained over three trials, with the greatest value obtained used for data analysis.

**Explanatory measures** will be made at baseline and outcome. Full training will be given to all trial center teams, with comprehensive monitoring and supervision arrangements. Data from all sites will be subject to prompt, rigorous quality control prior to statistical processing. All participants will be provided with full explanations and opportunities to ask questions, plenty of time to be made comfortable, and plenty of time to practice the tasks.

Structural brain imaging will be conducted in all patients in the same location during the same session.

First, a *T*_1_-weighted, 1 × 1 × 1-mm whole-brain image will be obtained using structural magnetic resonance imaging (MRI). Automated normalization, segmentation, and lesion identification will be performed as previously described 19, using spm12 (http://www.fil.ion.ucl.ac.uk/spm/software/spm12). This approach has a high sensitivity for delineating brain lesions and identifying tissue classes, thereby being useful in atrophy and white matter disease. Outputs include normalized lesion maps as well as gray and white matter maps consisting of voxel-wise values representing gray or white matter density.

Second, a map of the static magnetic field will be generated to allow for offline correction of image distortions.

Third, diffusion tensor imaging (DTI) data will be acquired at 2 × 2 × 2 mm. A diffusion tensor model will be fitted to data at each voxel to allow for maps of diffusion parameters (fractional anisotropy, mean diffusivity, and eigenvalues) to be generated for each participant at each scanning session. Probabilistic tractography (http://www.fmrib.ox.ac.uk/fsl) will be used to generate pathways of interest across the group 19, in particular the corticospinal tract.

Fourth, dual-echo *T*_2_-weighted and proton-density whole-brain MRI scans will be acquired at 1 × 1 × 3 mm resolution to allow for accurate delimitation of stroke volumes and for detection of other pathological changes, such as white matter hyperintensities.

A key purpose of FST is to improve the production of appropriate force in different muscles to enhance grasping and manipulation of objects by the paretic hand. We will therefore use a grip force task for functional MRI (fMRI), which is able to detect changes in brain activity corresponding to a force as low as 0.01N. As all participants will be able to produce the beginnings of prehension, they will be able to perform the fMRI grip task. Participants will be scanned while performing isometric handgrips of between 20% and 50% of paretic hand maximum grip force as measured just prior to scanning. Participants will be trained in how to perform the task prior to scanning. The experiment will be conducted according to an event-related design (auditory cues given at interstimulus interval of 7 ± 2 seconds) with actual exerted force recorded 20.

Analysis of fMRI data will follow standard approaches using spm12. Single-participant results will include voxel-wise values for (a) magnitude of brain activity during handgrips and (b) how much brain activity is modulated by handgrip force (20%, 50%). These values are independent of one another and provide complementary information on how the brain is working to generate motor output. In addition, we will use dynamic causal modeling (DCM) 21 to measure (a) connectivity between brain regions (coupling parameters) during grip and (b) changes in connectivity between brain regions (bilinear parameters) with increasing grip force.

Single pulses of transcranial magnetic stimulation (TMS), using a standard figure-of-eight coil, will be given over the hand and arm areas of the primary motor cortex of the lesioned hemisphere and, when possible, the nonlesioned hemisphere. Electromyographic recordings of motor evoked potentials (MEPs) over both contralateral and ipsilateral extensor carpi radialis and biceps brachii muscles will allow for characterization of recruitment curves, which will be constructed by measuring the amplitude of the MEP at between 100% and 130% (where possible) of active motor threshold 20,22.

#### Economic evaluation

Costs and effects will be monitored at baseline and outcome in order to inform the design of a future Phase III study. A preliminary estimate of cost-effectiveness will also be made. Resource items to be monitored will include input by the research therapist, length of stay in the original admission and any subsequent readmission, and other health-care and non-health-care contacts. Levels of informal care will also be monitored. The EuroQoL-5D 23 will be the main measure of effect, as it can be used to estimate the quality-adjusted life year gain.

### Assessment of safety

Comprehensive details of the trial safety monitoring process can be found in the operational trial protocol (http://www.fastindicate.com). In addition to events that may be expected to occur in a trial of this type, pain and fatigue will be particularly monitored, as there is a possibility that either MPT or FST, carried out in addition to CPT, could be associated with overuse syndrome, expressed by experience of pain or fatigue. We will follow the Norwich Clinical Trials Unit system of documenting and reporting serious adverse events (http://www.nnuh.nhs.uk/Dept.asp?ID=681). Adverse events will be recorded from randomization to end of trial.

### Data monitoring body

Ethical approval has been provided by the National Research Ethics Service. The University of East Anglia is the trial sponsor. All necessary local R&D governance approval will be obtained for each center in advance of recruitment. This trial is registered with Current Controlled Trials (ISRCTN19090862) and the UK Clinical Research Network Study Portfolio (UKCRN ID 12967). Independent trial oversight will be provided by a trial steering committee and a data monitoring and ethics committee set up in adherence to the UK Medical Research Council Guidelines for Good Clinical Practice in Clinical Trials.

### Sample size

The clustered data structure (patients grouped according to therapist within each treatment group) is accounted for in the design and analysis 24. This sample size calculation is based on actual ARAT data from our previous early-phase trial 9. Assuming an intraclass correlation coefficient of 0·01 in both treatment arms and three centers with a separate therapist for each randomized arm, a sample size of 99 participants per group would have 80% power to detect a clinically important mean difference of 6·2 in ARAT change when data were analyzed using a two-sample *t*-test with Satterthwaite correction, applying a 5% two-sided significance level and allowing for potentially different standard deviations in the CPT + MPT (7·9) and CPT + FST (19·3) groups. To account for clustering in the design (participants grouped according to therapist within each randomized treatment at each study site), a sample size inflation factor, 1 + (*m*-1)*ICC, is applied, where *m* is the cluster size and ICC is the intraclass correlation coefficient. Assuming that recruitment is evenly distributed across therapists, sample size is therefore inflated to 129 evaluable participants per group. The corresponding mean differences in ARAT change that would be detectable in a study of this size for ICCs of 0·02 and 0·03 would be 7·0 and 7·8 respectively, showing that the design is fairly insensitive to assumptions about the ICC. Finally, to allow for an attrition rate of 10% [7% in our previous single-center trial 9], 144 participants per group will be recruited for a total sample size of 288.

For the explanatory measurements, our experience indicates that of those meeting the criteria for this proposed trial, at least 70% will consent to participate, and 90% of these will complete the measurements. Thus, we anticipate 181 sets of explanatory measurements.

### Statistical analysis

Data for all participants will be analyzed according to participants’ allocated group. A single formal analysis will take place at the end of the study. Interim data summaries will be made available to the independent data monitoring and ethics committee.

With regard to missing data, we shall aim to do the following 25: (1) implement strategies to limit the amount of missing data; (2) develop an understanding of the causes for data being missing and (3), based on this, decide what the main assumption about the mechanism of missing data is; (4) conduct a statistical analysis which accounts for this assumption; and (5) perform sensitivity analyses to assess whether the conclusions depend strongly on the validity of the main assumption about the mechanism of missingness.

### Clinical efficacy

Continuous outcome variables will be compared between treatment groups using a multilevel normal linear model. Change from baseline to outcomes (day 43; Fig. 1) will be modeled, adjusting for baseline value, time after stroke, and 9HPT score as patient-level covariates. Which therapist administered the treatment will be included in the model as a zero-mean random effect. We will test, by comparing the log-likelihood, whether a separate random-effect variance is required for therapists delivering each treatment arm or whether a pooled variance is sufficient. The treatment effect will be summarized using the adjusted mean difference and 95% confidence interval.

Secondary analyses will include sensitivity to incomplete follow-up, descriptive analysis at each time-point, statistical modeling of follow-up measures at six-months, and a per-protocol analysis. For safety analysis, the number and percentage of participants experiencing each prespecified category of adverse event will be summarized by treatment group.

### Neural correlates of response to CPT + FST and CPT + MPT

Our main intention with these analyses is to infer differences between correlations between neural explanatory measurements and CPT + FST and those between such measurements and CPT + MPT (objective 2). Associations will be investigated between change from baseline in clinical outcomes and change from baseline in each of the explanatory measurements. We will make every possible effort to record and adjust for potential confounding baseline variables, such as baseline motor score.

The relationship will be explored further via multilevel linear regression, which will include which therapist administered the treatment as a random effect., We will assess whether changes in neuroimaging and/or neurophysiological measures are strongly associated with clinical improvements in the individual participant and, in particular, whether such associations differ between the CPT + FST and CPT + MPT groups. We acknowledge the potential value of structural mean models (SMM)/causal inference (CI) and are aware of the additional complexity due to clustering in the design. Therefore, we will investigate an extension of SMM/CI as a potential exploratory analysis 26.

The relationship will be explored further via multilevel linear regression which will include therapist as a random effect. We will assess whether changes in neuroimaging and/or neurophysiological measures are strongly associated with clinical improvements in the individual participant and in particular whether such associations differ between the CPT+FST and CPT+MPT groups.

### Indicators of response to CPT + FST and CPT + MPT

Baseline measurements considered will be (a) TMS- and DTI-based measurements related to corticospinal system integrity, (b) normalized lesion maps, (c) voxel-wise measurements of gray and white matter density, (d) voxel-wise measurements of brain activity during hand grip and its modulation by changing force, and (e) clinical variables, including motor scores. An interaction term between treatment group and each variable from (a) to (e) in turn will be added to the normal linear model for ARAT used in the clinical efficacy analysis. Continuous baseline variables will be categorized as high or low, the cutpoint being at the median of the observed data. We will adjust for the time after stroke category. Statistical significance of the interaction term will be assessed and treatment effect calculated within each of the high and low sub-groups of the interaction variable.

Further analysis will develop a multiple regression model within each treatment group to predict change in ARAT clinical outcome using baseline measurements. This will determine the sub-set of baseline variables independently associated with treatment response and will allow for a different group of baseline predictors within each treatment group. Principal components analysis will be used to reduce dimensionality of the predictor variables while retaining a meaningful interpretation of the principal components.

### Trial funding

FAST INdiCATE is funded by the Efficacy and Mechanism Evaluation programme (http://www.eme.ac.uk). EME reference: 10/60/30

## Summary

This trial aims to determine clinical efficacy of CPT + FST and CPT + MPT for enhancement of upper limb motor function early after stroke, neuro-biomechanical correlates associated with clinical improvement, and which stroke survivors may be most likely to respond to FST and which to MPT. The results are expected to inform a subsequent definitive clinical trial and also to lead to advances in knowledge of how the upper limb recovers after stroke in response to well-characterized interventions.
